# Carbonic Anhydrase IX Promotes Human Cervical Cancer Cell Motility by Regulating PFKFB4 Expression

**DOI:** 10.3390/cancers13051174

**Published:** 2021-03-09

**Authors:** Min-Chieh Hsin, Yi-Hsien Hsieh, Yi-Hsuan Hsiao, Pei-Ni Chen, Po-Hui Wang, Shun-Fa Yang

**Affiliations:** 1 Institute of Medicine, Chung Shan Medical University, Taichung 402, Taiwan; sinmusha@hotmail.com (M.-C.H.); hyhsien@csmu.edu.tw (Y.-H.H.); 54315@cch.org.tw (Y.-H.H.); peini@csmu.edu.tw (P.-N.C.); 2 Department of Medical Research, Chung Shan Medical University Hospital, Taichung 402, Taiwan; 3 School of Medicine, Chung Shan Medical University, Taichung 402, Taiwan; 4 Department of Obstetrics and Gynecology, Changhua Christian Hospital, Changhua 500, Taiwan; 5 Department of Obstetrics and Gynecology, Chung Shan Medical University Hospital, Taichung 402, Taiwan

**Keywords:** CAIX, PFKFB4, cervical cancer, metastasis

## Abstract

**Simple Summary:**

Carbonic anhydrase IX (CAIX) is a hypoxia-induced protein that is highly expressed in numerous human cancers. However, the molecular mechanisms involved in CAIX and human cervical cancer metastasis remain poorly understood. Our study found that CAIX overexpression increases PFKFB4 expression and EMT, promoting cervical cancer cell migration. CAIX could contribute to cervical cancer cell metastasis and its inhibition could be a cervical cancer treatment strategy.

**Abstract:**

Carbonic anhydrase IX (CAIX) is a hypoxia-induced protein that is highly expressed in numerous human cancers. However, the molecular mechanisms involved in CAIX and human cervical cancer metastasis remain poorly understood. In this study, CAIX overexpression in SiHa cells increased cell migration and epithelial-to-mesenchymal transition (EMT). Silencing CAIX in the Caski cell line decreased the motility of cells and EMT. Furthermore, the RNA-sequencing analysis identified a target gene, bifunctional 6-phosphofructo-2-kinase/fructose-2,6-bisphosphatase (PFKFB4), which is influenced by CAIX overexpression and knockdown. A positive correlation was found between CAIX expression and PFKFB4 levels in the cervical cancer of the TCGA database. Mechanistically, CAIX overexpression activated the phosphorylation of extracellular signal-regulated kinases (ERKs) to induce EMT and promote cell migration. In clinical results, human cervical cancer patients with CAIX^high^/PFKFB4^high^ expression in the late stage had higher rates of lymph node metastasis and the shortest survival time. Our study found that CAIX overexpression increases PFKFB4 expression and EMT, promoting cervical cancer cell migration. CAIX could contribute to cervical cancer cell metastasis and its inhibition could be a cervical cancer treatment strategy.

## 1. Introduction

Cervical cancer was the fourth most common cancer affecting women in 2018, following breast cancer, colorectal cancer, and lung cancer [1]. Developing countries have been affected by infectious agents such as human papillomavirus (HPV), which causes 70% of human cervical cancers [2,3]. Cervical cancer continues to be the second leading cause of mortality from cancer in women aged 20 to 39 years [4]. The hallmark of cancer is “metastasis”, which means the development of secondary tumors far from the original primary tumor [5]. Metastasis has been the key cause of the fatality rate of cancer, including human cervical cancer. The development of metastasis requires cancer cells to leave their main sites, circulate in the blood, withstand vascular pressure, adapt to the new cellular environment in secondary sites, and escape the deadly battle with immune cells [6,7]. Metastasis has been the primary cause of death for more than 90% of cancer patients [8]. Therefore, understanding the dynamics of this process will help determine targets for molecular therapies that may stop or may reverse cancer growth and metastasis. Dissemination of tumor cells is a multi-step process; epithelial-to-mesenchymal transition (EMT) occurs when tumor cells become more invasive, facilitated by a loss of cell–cell adhesion, and pass through the basement membrane into the blood or lymphatic system [9]. EMT is a process that enhances the capacity of migration and invasion and increases the expression of extracellular matrix (ECM) components [10].

Carbonic anhydrase IX (CAIX) is a membrane enzyme that catalyzes the reversible hydration of carbon dioxide, producing bicarbonate and hydrogen ions [11]. The CA family has 16 distinct members in humans. Among the CA members, CAIX is associated with cancer metastasis, progression, and therapeutic response [11,12,13]. Moreover, the catalytic activity of CAIX allows the intracellular pH (pHi) to be preserved in a range that is advantageous to cancer cell survival [14]. Among these CA isoforms, CAIX is unique because it is seldom expressed in normal tissues [15]. CAIX-inhibited is effective in anti-cancer has been demonstrated in several cancers [16,17,18,19]. Parkkila et al. showed that inhibitors of CAIX decreased renal cancer cell invasion [16]. In human breast cancer, CAIX-inhibited may reduce the ability of cell proliferation, migration, and invasion [17]. Furthermore, previous studies have demonstrated that CAIX can decrease the cell binding of E-cadherin to cytoskeleton and affect the metastatic ability of tumor cells [18,19]. Moreover, in several types of epithelial cancers, the high-level expression of CAIX was noted to be associated with such patient outcomes as breast cancer, non-small lung cancer, and cervical cancer [20,21,22,23]. A high level of CAIX is common in renal cell cancer, and CAIX target antibodies are being tested in phase III clinical trials (WX-G250, Rencarex^®^) [24]. CAIX has two major forms: one is a cell-associated, membranous form expressed in several types of cancer; the other is a soluble protein that may be released into a cell-culture medium or body fluids [25]. The soluble isoform of CAIX can be detected in the serum, so it can be used as an easy-to-use marker to stratify patients and monitor response [26]. In 1994, CAIX was cloned from HeLa cells, and it has been known that CAIX is the only tumor-associated member in the CA family [27]. Lieskovska et al. subsequently demonstrated that CAIX was involved in cell–cell and cell–matrix interactions [28]. In addition, due to the possible link between CAIX and HPV infection, it might be particularly meaningful to identify CA9 expression in cervical cancer cells [29]. The molecule may be used as a novel guiding principle for the diagnosis of HPV infection. 

Research on cancer metabolism has provided insights into the adaptive processes of cancer cells. The “Warburg effect”, also known as enhanced glycolysis, which provides the cancer cell with a metabolic basis for cell proliferation, is commonly observed in several cancer cells [30]. Glucose metabolism is regulated by four bifunctional 6-phosphofructo-2-kinase/fructose-2,6-bisphosphatase (PFKFB1, PFKFB2, PFKFB3, and PFKFB4) [31]. PFKFB4 is induced via hypoxia in several cancer cell lines. Moreover, PFKFB4 is overexpressed in the human breast, lung, colon, and pancreatic cancers compared with normal tissue in patients [31,32,33,34]. Several studies have indicated that PFKFB4 supports the survival of prostate cancer cells and glioma stem-like cells [35,36] but is not involved in normal cell survival. Therefore, PFKFB4 may be a potential target for the development of cancer therapeutics. Moreover, Chesney et al. demonstrated that PFKFB4 expression correlates with hypoxia. A significantly correlation was observed between PFKFB4 and CAIX expression in human lung adenocarcinoma xenografts [37]; however, how PFKFB4 is involved in cervical cancer is unclear at present.

## 2. Materials and Methods

### 2.1. Cell Lines and Culture

Human cervical cancer cell lines, SiHa, HeLa, C33A, and CC7T, were cultured in Dulbecco’s modified Eagle’s medium (Gibco) supplemented with 10% fetal bovine serum (FBS) (HyClone Laboratories) and 100 ng/mL each of streptomycin and penicillin (Sigma-Aldrich Corporation, St. Louis, MO, USA). The Caski cell line was cultured in an RPMI medium supplemented with 10% FBS and 100 ng/mL each of streptomycin and penicillin. All cell lines were cultured at 37 °C in a humidified atmosphere of 5% CO_2_.

### 2.2. pENTER-PFKFB4 Transfection

SiHa and Caski cells were seeded into 6 cm plates. After being cultured overnight, 5 µg of the empty pENTER-vector (GenDiscovery Biotechnology, Taipei, Taiwan) or pENTER-PFKFB4 were transfected into the cells and left for 6 h before carefully removing the reagent and culturing the cells with a fresh medium overnight.

### 2.3. Establishment of a Stable SiHa Cell Line Overexpressing CAIX

The cDNA of CAIX was amplified using PCR and cloned into the pcDNA3.0 vector. SiHa cells were transfected with the pcDNA3.0 vector or pcDNA3.0-CAIX using Lipofectamine^TM^ 2000 (Invitrogen, Carlsbad, CA, USA). After G418 selection for 21 days, only the CAIX overexpression vector or the stable clones with CAIX overexpression were obtained.

### 2.4. CAIX siRNA Transfection

Caski cells were seeded onto 6 cm plates. After being cultured overnight, we transfected the CAIX siRNA (S2270, Thermo Fisher Scientific, Waltham, MA, USA) into cells and working for 48 h at 37 °C. The effects of siRNA were detected by Western blot assay. 

### 2.5. Western Blot Assay

Total cell lysates were collected as preciously described [38]. The lysates were then incubated overnight at 4 °C with the primary antibodies and then with the secondary antibodies for 1 h at room temperature. Antibodies used were as follows: anti-CAIX (#5649, Cell Signaling), anti-p-ERK (#4370, Cell Signaling), anti-ERK (#9102, Cell Signaling), anti-E-cadherin (610182, BD), anti-vimentin (sc-6260, Santa Cruz), anti-PFKFB4 (GTX 107755, GeneTex), anti-p-MEK (#9121, Cell Signaling), anti-p-C-Raf (#9427, Cell Signaling), anti-C-Raf (#9422, Cell Signaling), and anti-actin (ab8226, Abcam). 

### 2.6. Cell Migration Assay

We harvested the cells using trypsin–ethylenediaminetetraacetic acid (EDTA) (Gibco), and the in vitro tumor metastasis assay was performed using a Boyden chamber (Neuro Probe). The cells were resuspended in a 10% FBS medium and loaded into the well on the upper part of the chamber and incubated for 24 h (migration) or 48 h (invasion) at 37 °C as preciously described [39]. 

### 2.7. Cell Proliferation Assay

A MTT colorimetric assay was performed to determine cell proliferation. Cells were seeded into 24-well plates and incubated at 37 °C for 1–5 days. The cells were then treated with MTT (5 mg/mL; Sigma) for 4 h at 37 °C. 

### 2.8. Immunofluorescence Staining

Cervical cancer cells were seeded in 24-well plates. After being incubated overnight, the cells were washed with 1× cold PBS (pH 7.4) twice. Samples were incubated with 4% paraformaldehyde as preciously described [38]. The samples were washed with 1× PBS for 3 min/per wash. The results were analyzed through immunofluorescence microscopy. 

### 2.9. Statistical Analyses

Statistically significant differences were set at *p* < 0.05 via Student’s *t*-test (SigmaPlot 10.0, San Jose, CA, USA). The values provided are the means ± SD of at least three independent experiments.

## 3. Results

### 3.1. Neither CAIX Overexpression in the SiHa Cell Line nor Its Knockdown in the Caski Cell Line Affects Cell Proliferation or the Cell Cycle

**Figure 1:**
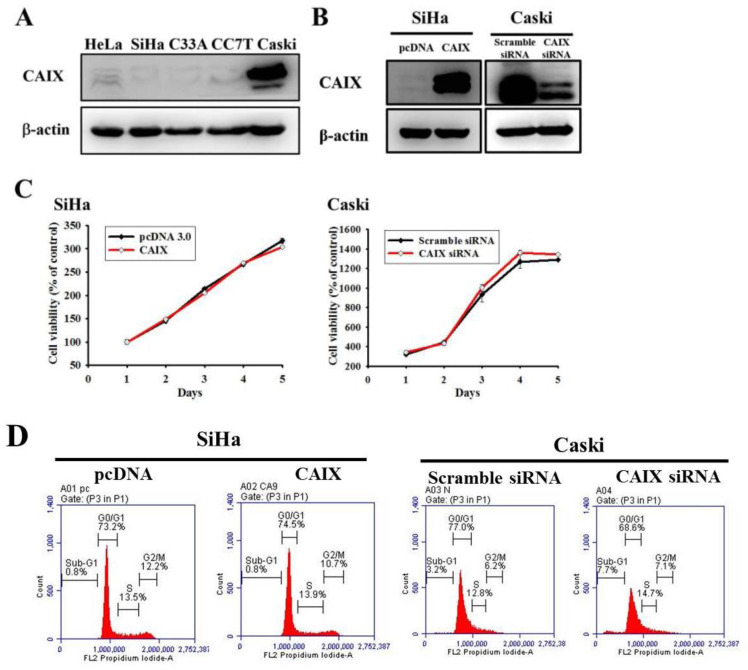
CAIX overexpression and knockdown in SiHa and Caski cell lines, respectively. (**A**) Endogenous CAIX protein levels were detected using Western blot analysis in human cervical cancer cell lines. (**B**) SiHa cells expressing low CAIX were transfected with a vector control (pcDNA 3.0) or CAIX-expressed vector (pcDNA-CAIX) for generating stably transfected clones (SiHa/pcDNA and SiHa/CAIX) after G418 selection. Caski cells expressing high CAIX were transiently transfected with scramble siRNA or CAIX siRNA. CAIX protein levels were detected through Western blot. (**C**) Growth curves of SiHa and Caski cells with or without CAIX expression was examined using the MTT assay. (**D**) Cell cycle profiles of transfected SiHa and Caski cells were determined using flow cytometry.

The relative CAIX expression in five human cervical cancer cell lines was evaluated (Figure 1A). Lower CAIX protein expressions in HeLa, SiHa, C33A and CC7T cells and the highest expressions of CAIX in Caski cell lines were observed. Accordingly, we chose SiHa (lower CAIX expression) and Caski (higher CAIX expression) cells in all subsequent experiments to investigate the role of CAIX in cercial cancer. Stable CAIX transfection was established in the SiHa cell line resulting in CAIX overexpression, whereas the knockdown of CAIX expression in the Caski cell line resulted in the silencing of CAIX (Figure 1B). The growth curve and cell cycle showed that neither CAIX overexpression nor knockdown affected the cell proliferation rate when compared with vector control cells (Figure 1C,D, respectively).

### 3.2. CAIX Expression Influences Cell Migration via EMT in Cervical Cancer Cell Lines

**Figure 2:**
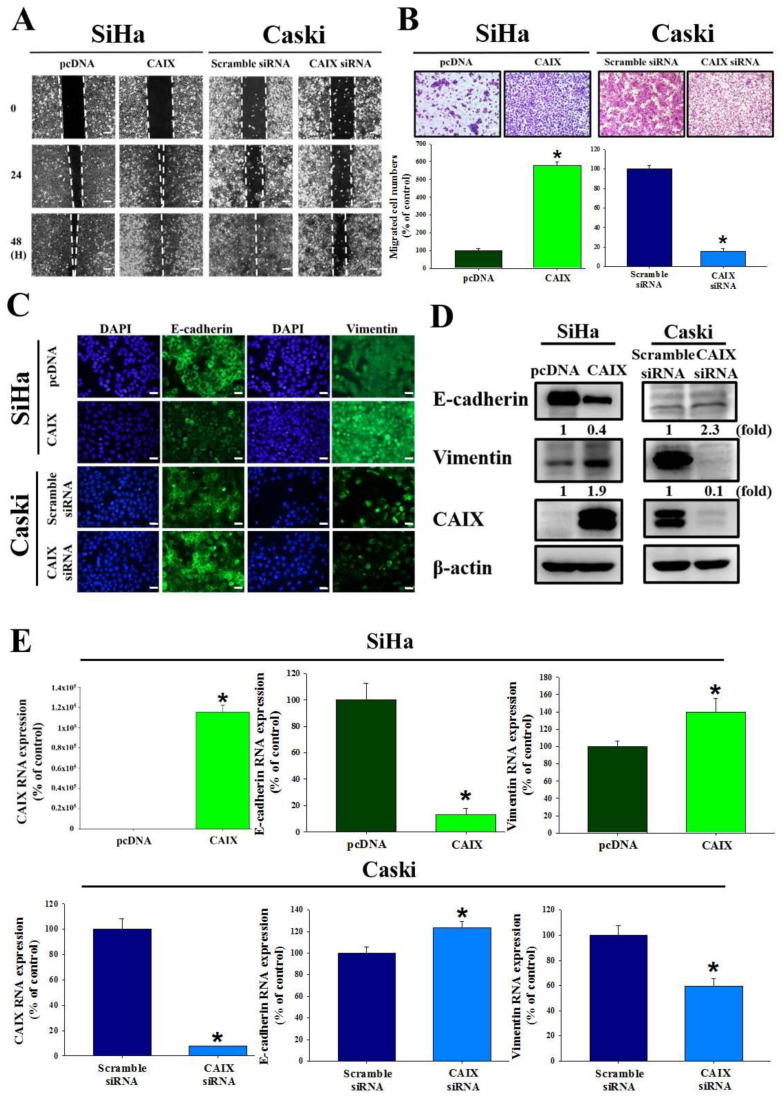
CAIX regulated cell migration and epithelial–mesenchymal transition in SiHa and Caski cells. (**A**) After CAIX overexpression or CAIX-silencing, cell motility was assessed by the wound healing assay and (**B**) Boyden chamber assay. Scale bar, 100 μm. (**C**) EMT proteins were detected using immunofluorescence staining. Scale bar, 50 μm. (**D**) The protein levels of E-cadherin and vimentin were detected by using Western blot assay. (**E**) The mRNA levels of E-cadherin and vimentin were detected by using real-time PCR. (*, *p* < 0.05).

The relationship between CAIX and the ability of SiHa and Caski cells to migrate and invade were investigated. We used a wound healing assay and the Boyden chamber assay to observe that CAIX overexpression significantly promoted migration in the SiHa cell line. Furthermore, the migratory ability decreased when CAIX expression was knocked down in the Caski cells (Figure 2A,B). Immunofluorescence staining showed that the morphological changes in CAIX-overexpressed SiHa cells were accompanied by reduced E-cadherin and increased vimentin levels. The Caski cells that had CAIX knockdown had increased E-cadherin expression (2.3-fold) and reduced vimentin expression (0.4-fold) (Figure 2C). In addition, the protein expression and RNA levels of E-cadherin and vimentin suggested that CAIX influenced EMT (Figure 2D,E). Therefore, reduced CAIX expression may inhibit the ability for cervical cancer cells to invade via EMT.

### 3.3. CAIX Mediates Cell Migration via Regulation of PFKFB4 Levels and EMT Protein in Cervical Cancer Cell Lines

**Figure 3:**
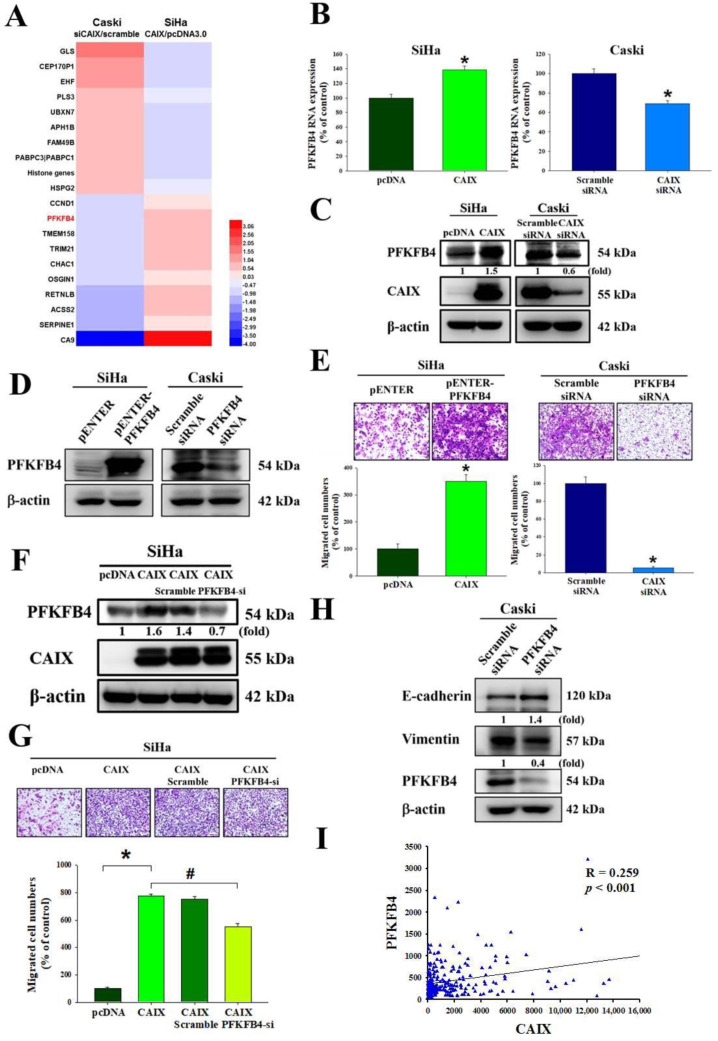
PFKFB4 is involved in CAIX-regulated cervical cancer migration. (**A**) Summary of mRNA array for CAIX overexpression in the SiHa cell line or CAIX-silencing in the Caski cell line according to log2 fold change. (**B**,**C**) The protein level and RNA level were detected by real-time PCR and Western blot. (**D**,**E**) After PFKFB4 overexpression in the SiHa cells or silencing in the Caski cells, Western blot and Boyden chamber assay were used for analysis. Scale bar, 100 μm. (**F**,**G**) After PFKFB4 knockdown in SiHa with stable CAIX overexpression, Boyden chamber assay and Western blot were used for analysis. Scale bar, 100 μm. (**H**) PFKFB4 knockdown in Caski cells and analysis by Western blot. (**I**) An association between CAIX and PFKFB4 mRNA levels in the TCGA database. (*, *p* < 0.05, compared to pcDNA or Scramble siRNA; #, *p* < 0.05, compared to CAIX).

To identify the target genes regulated by CAIX, we performed mRNA microarray analyses of the SiHa cell line with stable CAIX overexpression in the pcDNA 3.0 vector or an empty vector as a negative control, and of Caski cells with CAIX silencing normalized to scramble siRNA as the negative control. As shown in Figure 3A and Table S1, some genes were upregulated, and others were downregulated via CAIX expression, with PFKFB4 levels significantly changing. RNA and protein levels verified that PFKFB4 was upregulated with CAIX overexpression and downregulated with CAIX knockdown (Figure 3B,C). The relationship between PFKFB4 and the migration ability of SiHa and Caski cells was investigated. Thus, PFKFB4 was then overexpressed in the SiHa cells and knocked down in Caski cells (Figure 3D). The results indicate that PFKFB4 may regulate the migratory ability of cervical cancer cell lines (Figure 3E). The migratory ability of SiHa cells after CAIX overexpression and PFKFB4 knockdown was also analyzed, and the results showed that silencing PFKFB4 repressed CAIX-induced migration but did not affect CAIX protein expression (Figure 3F,G), indicating that PFKFB4 acts downstream of CAIX. These results suggest that CAIX-mediated PFKFB4 expression is involved in CAIX-mediated cell motility. Furthermore, we found that knocking down PFKFB4 in the Caski cell line may influence EMT protein expression, increasing E-cadherin and decreasing vimentin (Figure 3H). Moreover, analysis of The Cancer Genome Atlas (TCGA) database showed a correlation between CAIX and PFKFB4 (*p* < 0.001, R = 0.2259) in human cervical cancer (Figure 3I). 

### 3.4. CAIX Overexpression Activates ERK Phosphorylation to Induce EMT and Promote Cell Migration

**Figure 4:**
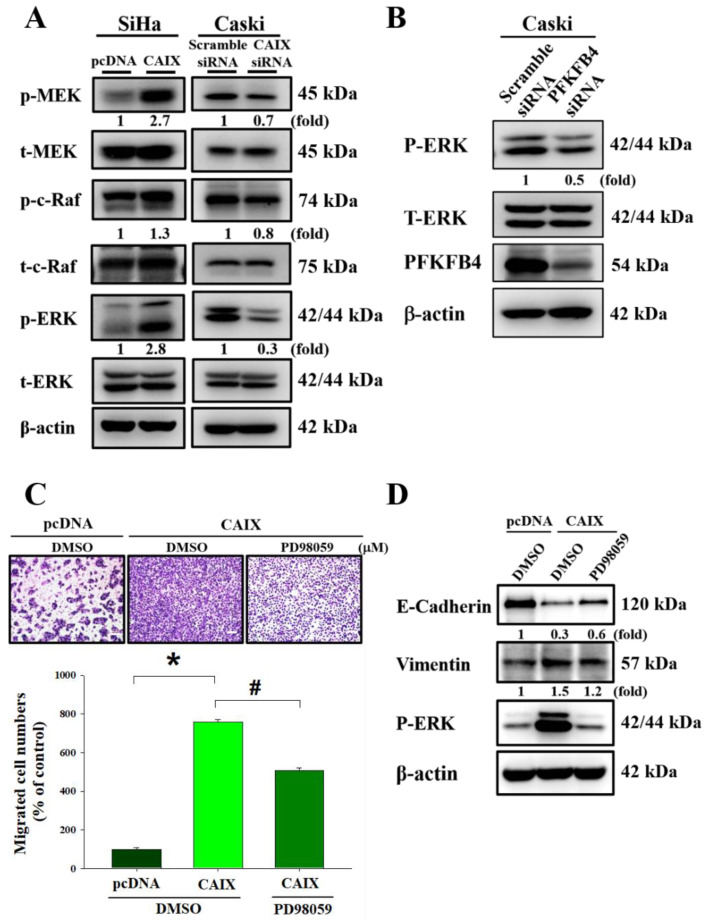
The MEK/Raf/ERK signaling pathways are crucial for CAIX-induced cell migration and EMT. (**A**) The levels of total and phosphorylated MEK, Raf, and ERK1/2 in SiHa and Caski cells with or without CAIX expression were determined using Western blot analyses. b-Actin was used as a loading control. (**B**) After knockdown PFKFB4 expression, total and phosphorylated ERK1/2 expression was determined using Western blot analyses. (**C**,**D**) Boyden chamber assay and Western blot was used to analyze the migration and protein level of PD98059 treating in SiHa cells with CAIX overexpression. Scale bar, 100 μm. (*, *p* < 0.05, compared to pcDNA or Scramble siRNA; #, *p* < 0.05, compared to CAIX).

To determine the signaling pathways involved in the CAIX-regulated migratory ability of cervical cancer cells, we measured the expression of the MAPK signaling pathway in cells with CAIX overexpression and cells with CAIX knockdown. Figure 4A shows that MEK, c-Raf, and ERK1/2 phosphorylation was increased in SiHa cells with CAIX overexpression, whereas MEK, c-Raf, and ERK1/2 phosphorylation was decreased in Caski cells that had CAIX silencing. Furthermore, PFKFB4 knockdown decreased ERK1/2 phosphorylation in Caski cells (Figure 4B). In addition, SiHa cells that had stable CAIX expression were treated with the MAPK kinase inhibitor PD98059. ERK inhibition reversed the CAIX-induced EMT and cell migration in the SiHa cell line (Figure 4C,D). These data show that CAIX regulates cell migration and EMT expression via the MAPK-ERK pathway. 

### 3.5. Human Clinical Late-Stage Cervical Cancer Patients with CAIX^high^/PFKFB4^high^ Expression Have Lymph Node Metastasis and the Shortest Survival Time

**Figure 5:**
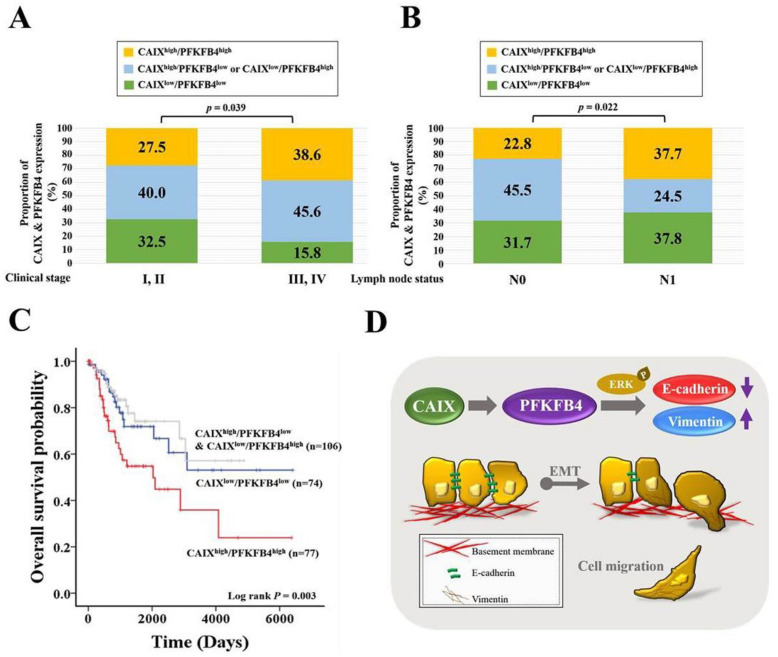
Clinical late-stage and lymph node metastasis are with CAIX^high^/PFKFB4^high^ in cervical cancer patients. (**A**) The association of the combined expression of CAIX and PFKFB4 with the clinical stage (I–IV) or (**B**) lymph node metastasis (N0–N1) in patients with cervical cancer patients. Grouped by CAIX^high^/PFKFB4^high^, CAIX^high^/PFKFB4^low^ or CAIX^low^/PFKFB4^high^, and CAIX^low^/PFKFB4^low^. (**C**) Kaplan–Meier curves for overall cervical cancer patient survival, grouped by CAIX^high^/PFKFB4^high^, CAIX^high^/PFKFB4^low^ or CAIX^low^/PFKFB4^high^, and CAIX^low^/PFKFB4^low^. (**D**) Schematic summary of the CAIX-PFKFB4-EMT signaling pathway.

To determine the association between CAIX and PFKFB4 expression in human cervical cancer, we analyzed the mRNA expression of CAIX and PFKFB4 in data sets from TCGA and the Gene Expression Omnibus database (GSE29570 and GSE52903). As shown in Figure 5A,B, the cervical cancer patients with a high CAIX level and high PFKFB4 level (CAIX^high^/PFKFB4^high^) were more predisposed to advanced clinical stage and lymph node metastasis than those expressing CAIX^low^/PFKFB4^low^, CAIX^high^/PFKFB4^low^, and CAIX^low^/PFKFB4^high^. Most importantly, the patients with CAIX^high^/PFKFB4^high^ had the shortest survival time compared with patients who had CAIX^low^/PFKFB4^low^, CAIX^high^/PFKFB4^low^, or CAIX^low^/PFKFB4^high^ (Figure 5C).

## 4. Discussion

Human cervical cancer is one of the most common gynecological malignancies. Lymph node metastasis is one of the leading factors of poor prognosis in cervical cancer [40]; however, the mechanisms involved in cervical cancer metastasis remain elusive. Numerous studies have provided evidence of a role for CAIX in cell migration, invasion, growth, and metastasis of tumors [41,42,43,44,45,46,47,48,49,50]. CAIX inhibition has been identified in preclinical studies that use various cancer models as being important in anticancer therapy, with some studies demonstrating that CAIX may affect the response of cancers to radiation [51,52]. The aims of our study were to figure out whether CAIX expression was a key factor in the motility of human cervical cancer cells, and which one downstream was regulated by CAIX. In human oral cancer cells, studies have determined that CAIX overexpression may induce cell motility by activating matrix metalloproteinase-9 [53]. In this study, we found that CAIX expression regulated epithelial–mesenchymal transition and cell migration in human cervical cancer cells (Figure 2). Fiaschi et al. proved that carbonic anhydrase IX drives epithelial-mesenchymal transition in prostate carcinoma cells [54]. The results can further corroborate our findings in this study. Although several studies have noted that many of the antitumor effects of CAIX inhibition might depend on pH regulation, some studies have determined that CAIX may interact with several signaling pathways involved in cellular responses to radiation. These findings indicate that pH-independent CAIX inhibition might also be important in tumor progression. In addition, we observed that CAIX increases cervical cell migration by upregulating PFKFB4 mRNA level and protein expression. This is the first finding that indicated a positive correlation between CAIX and PFKFB4 in human cervical cancer. PFKFB4 is a regulatory enzyme that synthesizes a stimulator of glycolysis, and it is highly expressed in several types of cancer and correlates with poor survival in breast cancer patients [55,56]. In past years, research on the metabolism of cancer cell had concentrated on investigating the single cell type. In recent studies, a more complicated situation in which metabolic heterogeneity in the tumor plays a key role in cancer progression has been demonstrated [57,58,59]. PFKFBs are encoded by four genes *(PFKFB1–4*). Among them, PFKFB3 and PFKFB4 protein expression is important in tumor cell proliferation and survival. Furthermore, in nasopharyngeal carcinoma and oral squamous cell carcinoma, PFKFB3 is crucial for metastasis [60,61]. Dasgupta et al. noted that PFKFB4 can phosphorylate the steroid receptor coactivator-3 (SRC-3) and lead to enhance ER co-activation and cell proliferation in human breast cancer [56]. In addition, one study indicated that PFKFB4 promotes breast cancer metastasis in a p38-dependent manner that initiates HAS2 transcription and expression [55]. In our study, we found that CAIX-PFKFB4 influenced cell migration by phosphorylating the MEK/Raf/ERK.

The upregulation of CAIX is probably an adaptation to aerobic glycolysis in tumor cells for the maintenance of intracellular pH in advanced carcinomas. The acidification of extracellular space by this mechanism may contribute to tumor cell invasion and the development of metastases [62,63]. We found that cervical cancer patients with CAIX^high^/PFKFB4^high^ have the shortest survival time (Figure 5C). In a recently published study, the expression of CAIX did not demonstrate a correlation with serum CAIX concentrations in renal cell cancer. It had been demonstrated that serum CAIX was associated with tumor size but not with intratumoral CAIX expression. Hypoxia in the tumor might be increased with tumor size, which will cause a soluble form of CAIX release into the bloodstream [64]. This study could explain why the survival rate of the CAIX^high^/PFKFB4^low^ & CAIX^low^/PFKFB4^high^ group was the highest in our study (Figure 5C). Moreover, we considered that, in terms of the survival rate of cervical cancer patients, some of the mechanisms and influences regulated by CAIX/PFKFB4 have not yet been discovered. Therefore, the impact of CAIX, PFKFB4, and clinicopathological characteristics of cervical cancer should be further investigated in the future. Nevertheless, the limitations of this study include the lack of an in vivo study and further studies are required to validate our findings. 

## 5. Conclusions

In summary, our present study highlights the importance of a signaling pathway, in cervical cancer, with a significant association between CAIX, PFKFB4, and the EMT proteins E-cadherin and vimentin. Moreover, we found that CAIX promotes cell migration by regulating PFKFB4 expression and increasing EMT. These findings provide new insights into the role of CAIX/PFKFB4 and the molecular mechanism involved in the progression of cervical cancer, suggesting that CAIX/PFKFB4 could be a potential diagnostic and therapeutic target in cervical cancer.

## Data Availability

Not applicable.

## Supplementary Materials

The following are available online at https://www.mdpi.com/2072-6694/13/5/1174/s1, Table S1: List of mRNA microarray identified genes upregulation and downregulation for CAIX overexpression in the SiHa cell line and CAIX-silencing in the Caski cell line. Figure S1: The original blot images of Figures.

## References

[1] Arbyn M., Weiderpass E., Bruni L., de Sanjose S., Saraiya M., Ferlay J., Bray F. (2020). Estimates of incidence and mortality of cervical cancer in 2018: A worldwide analysis. Lancet Glob. Health.

[2] Akinyemiju T.F. (2012). Socio-economic and health access determinants of breast and cervical cancer screening in low-income countries: Analysis of the world health survey. PLoS ONE.

[3] Munoz N., Bosch F.X., de Sanjose S., Herrero R., Castellsague X., Shah K.V., Snijders P.J., Meijer C.J., International Agency for Research on Cancer Multicenter Cervical Cancer Study Group (2003). Epidemiologic classification of human papillomavirus types associated with cervical cancer. N. Engl. J. Med..

[4] zur Hausen H. (2002). Papillomaviruses and cancer: From basic studies to clinical application. Nat. Rev. Cancer.

[5] Fares J., Fares M.Y., Khachfe H.H., Salhab H.A., Fares Y. (2020). Molecular principles of metastasis: A hallmark of cancer revisited. Signal. Transduct. Target. Ther..

[6] Maitra A. (2019). Molecular envoys pave the way for pancreatic cancer to invade the liver. Nature.

[7] Massague J., Obenauf A.C. (2016). Metastatic colonization by circulating tumour cells. Nature.

[8] Steeg P.S. (2006). Tumor metastasis: Mechanistic insights and clinical challenges. Nat. Med..

[9] Thews O., Riemann A. (2019). Tumor ph and metastasis: A malignant process beyond hypoxia. Cancer Metastasis Rev..

[10] Kalluri R., Weinberg R.A. (2009). The basics of epithelial-mesenchymal transition. J. Clin. Investig..

[11] Chafe S.C., Dedhar S. (2015). Carving out its niche: A role for carbonic anhydrase ix in pre-metastatic niche development. Oncoimmunology.

[12] McDonald P.C., Winum J.Y., Supuran C.T., Dedhar S. (2012). Recent developments in targeting carbonic anhydrase ix for cancer therapeutics. Oncotarget.

[13] Neri D., Supuran C.T. (2011). Interfering with ph regulation in tumours as a therapeutic strategy. Nat. Rev. Drug Discov..

[14] Chen Z., Ai L., Mboge M.Y., Tu C., McKenna R., Brown K.D., Heldermon C.D., Frost S.C. (2018). Differential expression and function of caix and caxii in breast cancer: A comparison between tumorgraft models and cells. PLoS ONE.

[15] Kaluz S., Kaluzova M., Liao S.Y., Lerman M., Stanbridge E.J. (2009). Transcriptional control of the tumor- and hypoxia-marker carbonic anhydrase 9: A one transcription factor (hif-1) show?. Biochim. Biophys. Acta.

[16] Parkkila S., Rajaniemi H., Parkkila A.K., Kivela J., Waheed A., Pastorekova S., Pastorek J., Sly W.S. (2000). Carbonic anhydrase inhibitor suppresses invasion of renal cancer cells in vitro. Proc. Natl. Acad. Sci. USA.

[17] Lou Y., McDonald P.C., Oloumi A., Chia S., Ostlund C., Ahmadi A., Kyle A., Auf dem Keller U., Leung S., Huntsman D. (2011). Targeting tumor hypoxia: Suppression of breast tumor growth and metastasis by novel carbonic anhydrase ix inhibitors. Cancer Res..

[18] Svastova E., Zilka N., Zat’ovicova M., Gibadulinova A., Ciampor F., Pastorek J., Pastorekova S. (2003). Carbonic anhydrase ix reduces e-cadherin-mediated adhesion of mdck cells via interaction with beta-catenin. Exp. Cell Res..

[19] Shin H.J., Rho S.B., Jung D.C., Han I.O., Oh E.S., Kim J.Y. (2011). Carbonic anhydrase ix (ca9) modulates tumor-associated cell migration and invasion. J. Cell Sci..

[20] Hussain S.A., Ganesan R., Reynolds G., Gross L., Stevens A., Pastorek J., Murray P.G., Perunovic B., Anwar M.S., Billingham L. (2007). Hypoxia-regulated carbonic anhydrase ix expression is associated with poor survival in patients with invasive breast cancer. Br. J. Cancer.

[21] Klatte T., Seligson D.B., Rao J.Y., Yu H., de Martino M., Kawaoka K., Wong S.G., Belldegrun A.S., Pantuck A.J. (2009). Carbonic anhydrase ix in bladder cancer: A diagnostic, prognostic, and therapeutic molecular marker. Cancer.

[22] Loncaster J.A., Harris A.L., Davidson S.E., Logue J.P., Hunter R.D., Wycoff C.C., Pastorek J., Ratcliffe P.J., Stratford I.J., West C.M. (2001). Carbonic anhydrase (ca ix) expression, a potential new intrinsic marker of hypoxia: Correlations with tumor oxygen measurements and prognosis in locally advanced carcinoma of the cervix. Cancer Res..

[23] Swinson D.E., Jones J.L., Richardson D., Wykoff C., Turley H., Pastorek J., Taub N., Harris A.L., O’Byrne K.J. (2003). Carbonic anhydrase ix expression, a novel surrogate marker of tumor hypoxia, is associated with a poor prognosis in non-small-cell lung cancer. J. Clin. Oncol..

[24] Chamie K., Klöpfer P., Bevan P., Störkel S., Said J., Fall B., Belldegrun A.S., Pantuck A.J. (2015). Carbonic anhydrase-ix score is a novel biomarker that predicts recurrence and survival for high-risk, nonmetastatic renal cell carcinoma: Data from the phase iii ariser clinical trial. Urol. Oncol..

[25] Zavada J., Zavadova Z., Zat’ovicova M., Hyrsl L., Kawaciuk I. (2003). Soluble form of carbonic anhydrase ix (ca ix) in the serum and urine of renal carcinoma patients. Br. J. Cancer.

[26] Woelber L., Kress K., Kersten J.F., Choschzick M., Kilic E., Herwig U., Lindner C., Schwarz J., Jaenicke F., Mahner S. (2011). Carbonic anhydrase ix in tumor tissue and sera of patients with primary cervical cancer. BMC Cancer.

[27] Opavsky R., Pastorekova S., Zelnik V., Gibadulinova A., Stanbridge E.J., Zavada J., Kettmann R., Pastorek J. (1996). Human mn/ca9 gene, a novel member of the carbonic anhydrase family: Structure and exon to protein domain relationships. Genomics.

[28] Lieskovska J., Opavsky R., Zacikova L., Glasova M., Pastorek J., Pastorekova S. (1999). Study of in vitro conditions modulating expression of mn/ca ix protein in human cell lines derived from cervical carcinoma. Neoplasma.

[29] Kim J.Y., Shin H.J., Kim T.H., Cho K.H., Shin K.H., Kim B.K., Roh J.W., Lee S., Park S.Y., Hwang Y.J. (2006). Tumor-associated carbonic anhydrases are linked to metastases in primary cervical cancer. J. Cancer Res. Clin. Oncol..

[30] Yao L., Wang L., Cao Z.G., Hu X., Shao Z.M. (2019). High expression of metabolic enzyme pfkfb4 is associated with poor prognosis of operable breast cancer. Cancer Cell Int..

[31] Minchenko O.H., Tsuchihara K., Minchenko D.O., Bikfalvi A., Esumi H. (2014). Mechanisms of regulation of pfkfb expression in pancreatic and gastric cancer cells. World J. Gastroenterol..

[32] Minchenko O.H., Ochiai A., Opentanova I.L., Ogura T., Minchenko D.O., Caro J., Komisarenko S.V., Esumi H. (2005). Overexpression of 6-phosphofructo-2-kinase/fructose-2,6-bisphosphatase-4 in the human breast and colon malignant tumors. Biochimie.

[33] Minchenko O.H., Ogura T., Opentanova I.L., Minchenko D.O., Ochiai A., Caro J., Komisarenko S.V., Esumi H. (2005). 6-phosphofructo-2-kinase/fructose-2,6-bisphosphatase gene family overexpression in human lung tumor. Ukr. Biokhim. Zh. (1999).

[34] Minchenko O., Opentanova I., Caro J. (2003). Hypoxic regulation of the 6-phosphofructo-2-kinase/fructose-2,6-bisphosphatase gene family (pfkfb-1-4) expression in vivo. FEBS Lett..

[35] Goidts V., Bageritz J., Puccio L., Nakata S., Zapatka M., Barbus S., Toedt G., Campos B., Korshunov A., Momma S. (2012). Rnai screening in glioma stem-like cells identifies pfkfb4 as a key molecule important for cancer cell survival. Oncogene.

[36] Ros S., Santos C.R., Moco S., Baenke F., Kelly G., Howell M., Zamboni N., Schulze A. (2012). Functional metabolic screen identifies 6-phosphofructo-2-kinase/fructose-2,6-biphosphatase 4 as an important regulator of prostate cancer cell survival. Cancer Discov..

[37] Chesney J., Clark J., Klarer A.C., Imbert-Fernandez Y., Lane A.N., Telang S. (2014). Fructose-2,6-bisphosphate synthesis by 6-phosphofructo-2-kinase/fructose-2,6-bisphosphatase 4 (pfkfb4) is required for the glycolytic response to hypoxia and tumor growth. Oncotarget.

[38] Su C.W., Chang Y.C., Chien M.H., Hsieh Y.H., Chen M.K., Lin C.W., Yang S.F. (2019). Loss of timp3 by promoter methylation of sp1 binding site promotes oral cancer metastasis. Cell Death Dis..

[39] Yang W.E., Ho Y.C., Tang C.M., Hsieh Y.S., Chen P.N., Lai C.T., Yang S.F., Lin C.W. (2019). Duchesnea indica extract attenuates oral cancer cells metastatic potential through the inhibition of the matrix metalloproteinase-2 activity by down-regulating the mek/erk pathway. Phytomedicine.

[40] Liu Z., Hu K., Liu A., Shen J., Hou X., Lian X., Sun S., Yan J., Zhang F. (2016). Patterns of lymph node metastasis in locally advanced cervical cancer. Medicine.

[41] Ward C., Meehan J., Mullen P., Supuran C., Dixon J.M., Thomas J.S., Winum J.Y., Lambin P., Dubois L., Pavathaneni N.K. (2015). Evaluation of carbonic anhydrase ix as a therapeutic target for inhibition of breast cancer invasion and metastasis using a series of in vitro breast cancer models. Oncotarget.

[42] Chiche J., Ilc K., Laferriere J., Trottier E., Dayan F., Mazure N.M., Brahimi-Horn M.C., Pouyssegur J. (2009). Hypoxia-inducible carbonic anhydrase ix and xii promote tumor cell growth by counteracting acidosis through the regulation of the intracellular ph. Cancer Res..

[43] Morris J.C., Chiche J., Grellier C., Lopez M., Bornaghi L.F., Maresca A., Supuran C.T., Pouyssegur J., Poulsen S.A. (2011). Targeting hypoxic tumor cell viability with carbohydrate-based carbonic anhydrase ix and xii inhibitors. J. Med. Chem..

[44] Doyen J., Parks S.K., Marcie S., Pouyssegur J., Chiche J. (2012). Knock-down of hypoxia-induced carbonic anhydrases ix and xii radiosensitizes tumor cells by increasing intracellular acidosis. Front. Oncol..

[45] Beltran A.S., Russo A., Lara H., Fan C., Lizardi P.M., Blancafort P. (2011). Suppression of breast tumor growth and metastasis by an engineered transcription factor. PLoS ONE.

[46] McIntyre A., Patiar S., Wigfield S., Li J.L., Ledaki I., Turley H., Leek R., Snell C., Gatter K., Sly W.S. (2012). Carbonic anhydrase ix promotes tumor growth and necrosis in vivo and inhibition enhances anti-vegf therapy. Clin. Cancer Res..

[47] Lock F.E., McDonald P.C., Lou Y., Serrano I., Chafe S.C., Ostlund C., Aparicio S., Winum J.Y., Supuran C.T., Dedhar S. (2013). Targeting carbonic anhydrase ix depletes breast cancer stem cells within the hypoxic niche. Oncogene.

[48] Meehan J., Ward C., Turnbull A., Bukowski-Wills J., Finch A.J., Jarman E.J., Xintaropoulou C., Martinez-Perez C., Gray M., Pearson M. (2017). Inhibition of ph regulation as a therapeutic strategy in hypoxic human breast cancer cells. Oncotarget.

[49] Swayampakula M., McDonald P.C., Vallejo M., Coyaud E., Chafe S.C., Westerback A., Venkateswaran G., Shankar J., Gao G., Laurent E.M.N. (2017). The interactome of metabolic enzyme carbonic anhydrase ix reveals novel roles in tumor cell migration and invadopodia/mmp14-mediated invasion. Oncogene.

[50] Radvak P., Repic M., Svastova E., Takacova M., Csaderova L., Strnad H., Pastorek J., Pastorekova S., Kopacek J. (2013). Suppression of carbonic anhydrase ix leads to aberrant focal adhesion and decreased invasion of tumor cells. Oncol. Rep..

[51] Koukourakis M.I., Giatromanolaki A., Sivridis E., Simopoulos K., Pastorek J., Wykoff C.C., Gatter K.C., Harris A.L. (2001). Hypoxia-regulated carbonic anhydrase-9 (ca9) relates to poor vascularization and resistance of squamous cell head and neck cancer to chemoradiotherapy. Clin. Cancer Res..

[52] Koukourakis M.I., Bentzen S.M., Giatromanolaki A., Wilson G.D., Daley F.M., Saunders M.I., Dische S., Sivridis E., Harris A.L. (2006). Endogenous markers of two separate hypoxia response pathways (hypoxia inducible factor 2 alpha and carbonic anhydrase 9) are associated with radiotherapy failure in head and neck cancer patients recruited in the chart randomized trial. J. Clin. Oncol..

[53] Yang J.S., Lin C.W., Hsieh Y.H., Chien M.H., Chuang C.Y., Yang S.F. (2017). Overexpression of carbonic anhydrase ix induces cell motility by activating matrix metalloproteinase-9 in human oral squamous cell carcinoma cells. Oncotarget.

[54] Fiaschi T., Giannoni E., Taddei M.L., Cirri P., Marini A., Pintus G., Nativi C., Richichi B., Scozzafava A., Carta F. (2013). Carbonic anhydrase ix from cancer-associated fibroblasts drives epithelial-mesenchymal transition in prostate carcinoma cells. Cell Cycle.

[55] Gao R., Liu Y., Li D., Xun J., Zhou W., Wang P., Liu C., Li X., Shen W., Su W. (2018). Pfkfb4 promotes breast cancer metastasis via induction of hyaluronan production in a p38-dependent manner. Cell. Physiol. Biochem..

[56] Dasgupta S., Rajapakshe K., Zhu B., Nikolai B.C., Yi P., Putluri N., Choi J.M., Jung S.Y., Coarfa C., Westbrook T.F. (2018). Metabolic enzyme pfkfb4 activates transcriptional coactivator src-3 to drive breast cancer. Nature.

[57] Sonveaux P., Vegran F., Schroeder T., Wergin M.C., Verrax J., Rabbani Z.N., De Saedeleer C.J., Kennedy K.M., Diepart C., Jordan B.F. (2008). Targeting lactate-fueled respiration selectively kills hypoxic tumor cells in mice. J. Clin. Investig..

[58] Martinez-Outschoorn U., Sotgia F., Lisanti M.P. (2014). Tumor microenvironment and metabolic synergy in breast cancers: Critical importance of mitochondrial fuels and function. Semin. Oncol..

[59] Martinez-Outschoorn U.E., Peiris-Pages M., Pestell R.G., Sotgia F., Lisanti M.P. (2017). Cancer metabolism: A therapeutic perspective. Nat. Rev. Clin. Oncol..

[60] Li H.M., Yang J.G., Liu Z.J., Wang W.M., Yu Z.L., Ren J.G., Chen G., Zhang W., Jia J. (2017). Blockage of glycolysis by targeting pfkfb3 suppresses tumor growth and metastasis in head and neck squamous cell carcinoma. J. Exp. Clin. Cancer Res..

[61] Gu M., Li L., Zhang Z., Chen J., Zhang W., Zhang J., Han L., Tang M., You B., Zhang Q. (2017). Pfkfb3 promotes proliferation, migration and angiogenesis in nasopharyngeal carcinoma. J. Cancer.

[62] Svastova E., Hulikova A., Rafajova M., Zat’ovicova M., Gibadulinova A., Casini A., Cecchi A., Scozzafava A., Supuran C.T., Pastorek J. (2004). Hypoxia activates the capacity of tumor-associated carbonic anhydrase ix to acidify extracellular ph. FEBS Lett..

[63] Swietach P., Patiar S., Supuran C.T., Harris A.L., Vaughan-Jones R.D. (2009). The role of carbonic anhydrase 9 in regulating extracellular and intracellular ph in three-dimensional tumor cell growths. J. Biol. Chem..

[64] Zhou G.X., Ireland J., Rayman P., Finke J., Zhou M. (2010). Quantification of carbonic anhydrase ix expression in serum and tissue of renal cell carcinoma patients using enzyme-linked immunosorbent assay: Prognostic and diagnostic potentials. Urology.

