# Supplementary material for: Carbonic Anhydrase IX Promotes Human Cervical Cancer Cell Motility by Regulating PFKFB4 Expression
Source: Cancers (Basel). 2021 Mar 9;13(5):1174. doi: 10.3390/cancers13051174 (PMC7967120; doi:10.3390/cancers13051174)

Carbonic anhydrase IX promotes human cervical cancer cell motility by regulating PFKFB4 expression

Min-Chieh Hsin[1,2], Yi-Hsien Hsieh [1,2], Yi-Hsuan Hsiao[1,3,4], Pei-Ni Chen [1,2], Po-Hui Wang[1,3,5,*], and Shun-Fa Yang[1,2,*]

[1]Institute of Medicine, Chung Shan Medical University, Taichung 402, Taiwan; sinmusha@hotmail.com (M.C.H.); hyhsien@csmu.edu.tw (Y.H.H.); 54315@cch.org.tw (Y.H.H.); peini@csmu.edu.tw (P.N.C.); wang082160@gmail.com (P.H.W.)
[2]Department of Medical Research, Chung Shan Medical University Hospital, Taichung 402, Taiwan; ysf@csmu.edu.tw
[3]School of Medicine, Chung Shan Medical University, Taichung 402, Taiwan
[4]Department of Obstetrics and Gynecology, Changhua Christian Hospital, Changhua 500, Taiwan
[5]Department of Obstetrics and Gynecology, Chung Shan Medical University Hospital, Taichung 402, Taiwan
*Correspondence: wang082160@gmail.com (P.H.W.) and ysf@csmu.edu.tw (S.F.Y.)

Table S1. List of mRNA microarray identified genes upregulation and downregulation for CAIX overexpression in the SiHa cell line and CAIX-silencing in the Caski cell line.

**Gene symbol**	**Caski siCAIX/scramble Log2 (fold change)**	**SiHa CAIX/pcDNA3.0 Log2 (fold change)**
GLS	1.73512642103292	-0.521915467770771
CEP170P1	1.17684356410596	-0.567037824996468
EHF	1.03820776833435	-0.575610485652121
PLS3	0.867966045787852	-0.436274899151899
UBXN7	0.828502543242875	-0.477269305615549
APH1B	0.813555693876334	-0.480030100260504
FAM49B	0.726671671098768	-0.530662320369936
PABPC3/PABPC1	0.687738816669596	-0.533717248494197
Histone genes*	0.67895508734665	-0.545665204019582
HSPG2	0.659852366412425	-0.416535799774465
CCND1	-0.577961962283008	0.53325818128498
PFKFB4	-0.620813529189314	0.642339799306373
TMEM158	-0.660756841062532	0.549518132354652
TRIM21	-0.722670242612071	0.542338073785298
CHAC1	-0.751543587877312	0.597887631108036
OSGIN1	-0.979835289667423	0.418088831056177
RETNLB	-0.999069240049108	0.793512907154393
ACSS2	-1.08871707710427	0.576172665509403
SERPINE1	-1.24784413650027	0.535493380466762
CA9	-4.00385528804701	3.56711399351252

*Histone genes: HIST2H2BF, HIST1H2BN, HIST1H2BE, HIST1H2BK, HIST1H2BL, HIST2H2BC, HIST1H2BI, HIST2H2BA, HIST1H2BC, HIST1H2BJ, HIST1H2BD, HIST3H2BB, HIST1H2BO, HIST1H2BH, HIST1H2BM

Figure S1: The original blot images of Figures.

Figure 1A

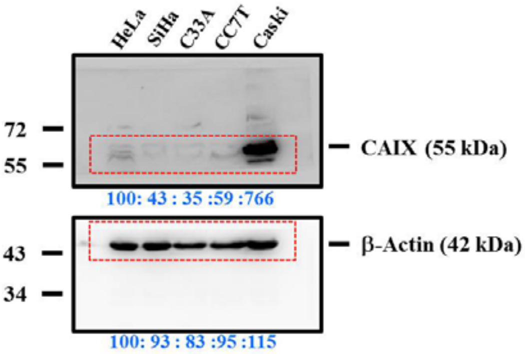

Figure 1B

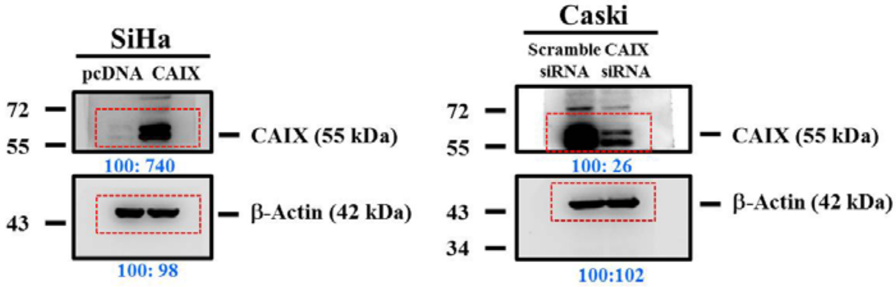

Figure 2D

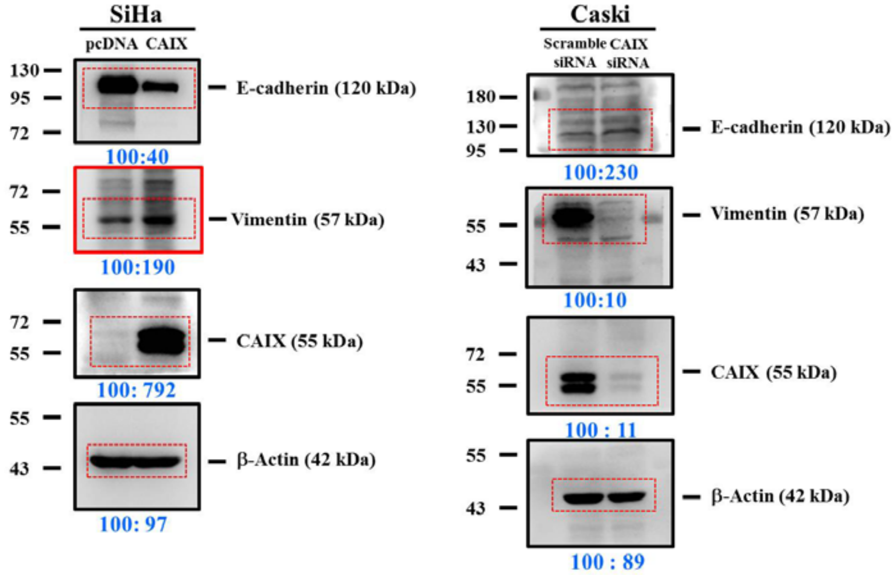

Figure 3C

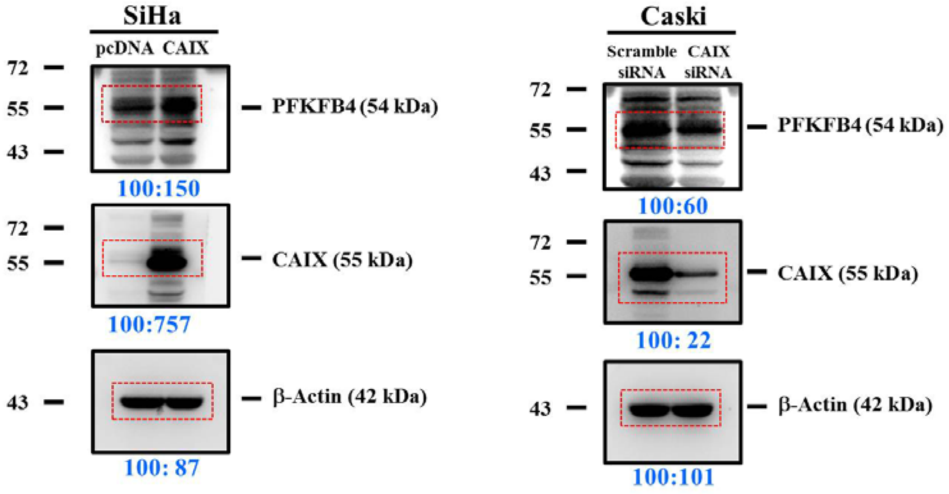

Figure 3D

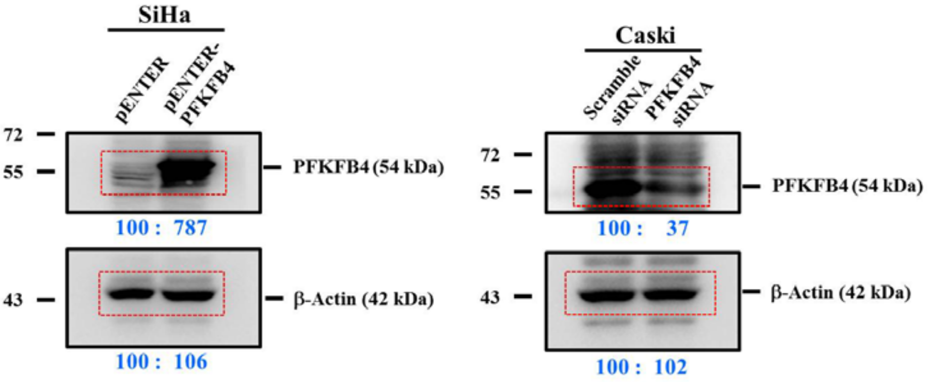

Figure 3F

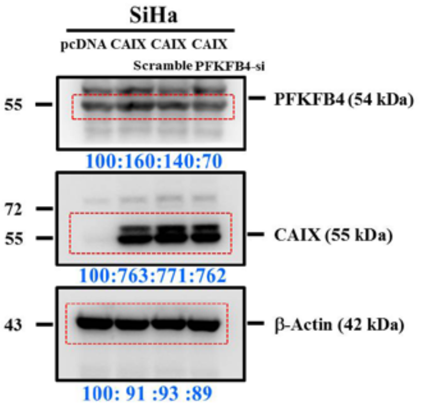

Figure 3H

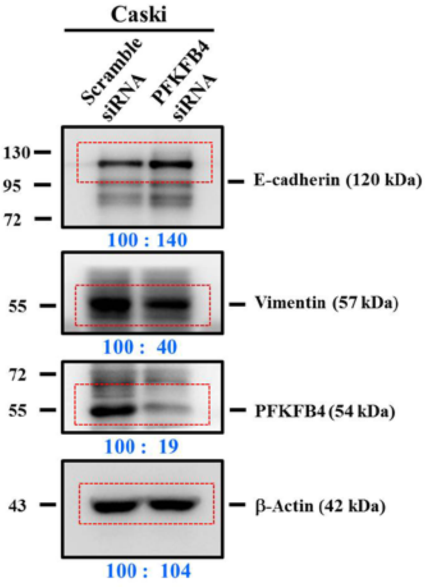

Figure 4A

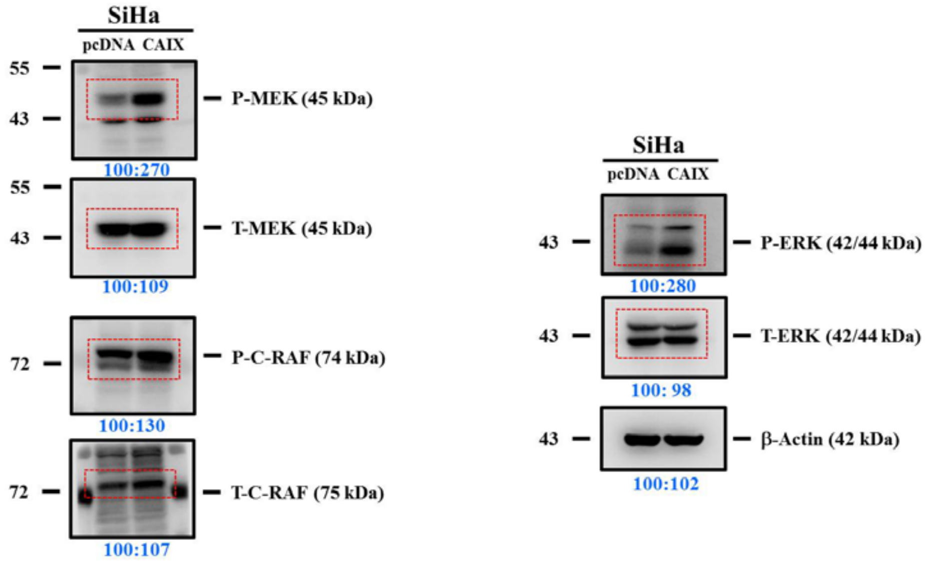

Figure 4A

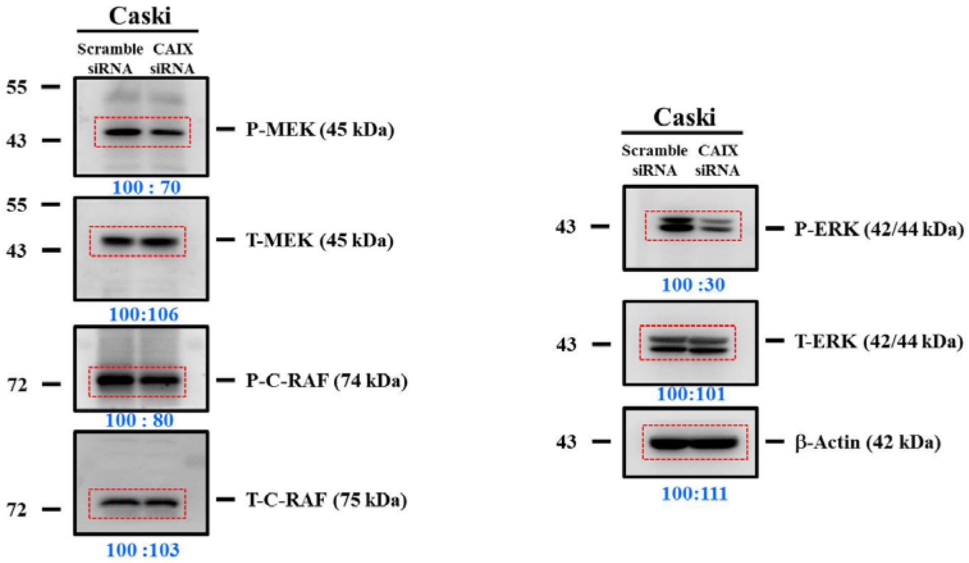

Figure 4B

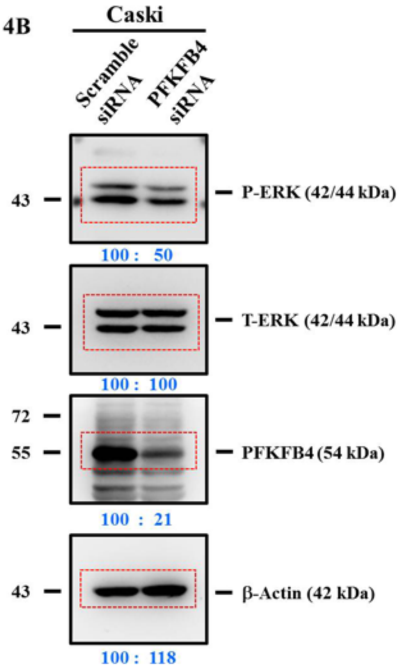

Figure 4D

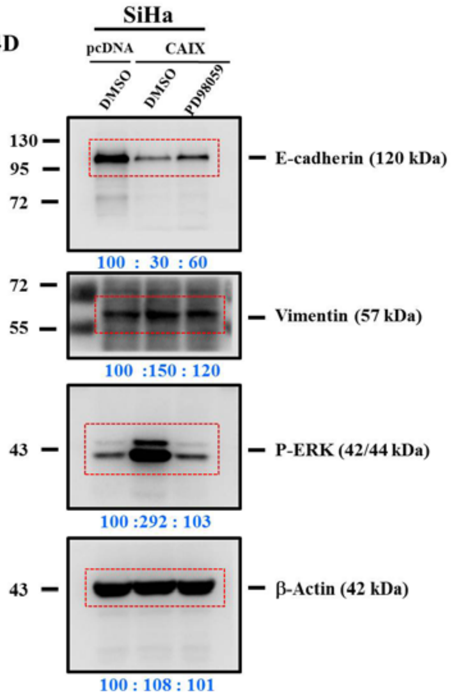

Supplement: Supplementary file 1 [file cancers-13-01174-s001.pdf]
